# The Impact of Flaxseed (*Linum usitatissimum* L.) Oil Supplementation on Human Health: A Human-Centric Evidence-Graded Approach

**DOI:** 10.3390/nu17111791

**Published:** 2025-05-25

**Authors:** Ying Nie, Yuchen Wang, Ju Hui, Danqing Shao, Ran Chen, Qianchun Deng, Yashu Chen, Xiangyu Wang, Dazhou Zhu

**Affiliations:** 1 Institute of Food and Nutrition Development, Ministry of Agriculture and Rural Affairs, Beijing 100081, China; nieying01@caas.cn (Y.N.); 82101232184@caas.cn (Y.W.); 2 Cofco Nutrition and Health Research Institute, Beijing 102209, China; huiju@cofco.com (J.H.); shaodanqing@cofco.com (D.S.); chenran@cofco.com (R.C.); 3 Oil Crops Research Institute, Chinese Academy of Agricultural Sciences, Wuhan 430062, China; dengqianchun@caas.cn (Q.D.); chenyashu@caas.cn (Y.C.)

**Keywords:** flaxseed oil, evidence evaluation, inflammation, blood lipid, blood pressure, insulin sensitivity

## Abstract

Background: Although flaxseed oil shows potential health benefits, there is a significant gap between preclinical studies (animal/in vitro) and actual effects in humans. The health benefits of flaxseed oil for humans remain unclear. Objective: This article seeks to precisely identify the health benefits of flaxseed oil by evaluating the scientific evidence from human trials on flaxseed oil. Methods: According to the gradation method of a human-centric evidence body, 13 articles were ultimately selected as evaluation evidence after applying inclusion/exclusion criteria to the 2148 papers retrieved from scholarly databases. Results: The evaluation results of the evidence body on inflammatory cytokines, blood pressure, insulin sensitivity, and blood lipid are all B. Additionally, the influences of flaxseed oil consumption on waist circumstance, mood, and cognition are still unclear. Conclusions: The results reveal that flaxseed oil intervention significantly decreases inflammatory cytokines, blood pressure, and insulin sensitivity, but does not affect blood lipid improvement. Meanwhile, the effect of flaxseed oil intervention on waist circumstance, mood, and cognition need more human trials to determine.

## 1. Introduction

With the increasing incidence of non-communicable diseases mainly caused by unhealthy diets and inevitable side effects of medication, researchers began to explore the effects of functional foods on human health. Flaxseed (*Linum usitatissimum* L.) is an ancient commercial oilseed crop with a variety of health benefits due to its rich content of *n*-3 polyunsaturated fatty acids, lignans, and dietary fiber [1]. Flaxseed can be processed into whole flaxseed powder or defatted flaxseed meal, with the oil further refined into cooking oil or capsules [2,3]. Edible flaxseed oil is extracted from dehulled flaxseed with mechanical pressing and refining to minimize nutrient loss [3]. Flaxseed oil comprises 73% of polyunsaturated fatty acids, 8% of saturated fatty acids, and 19% of monounsaturated fatty acids [4]. Remarkably, flaxseed oil contains 39–60% α-linolenic acid, with a beneficial *n*-6 to *n*-3 fatty acid ratio of 0.3:1 [5]. α-linolenic acid is an essential fatty acid that cannot be synthesized by the human body. Dietary sources like plant seeds, nuts, and marine products are necessary to provide enough ALA, which can convert to biologically active long-chain *n*-3 polyunsaturated fatty acid eicosatetraenoic acid (EPA; C20:5, *n*-3) and docosahexaenoic acid (DHA; C22:6, *n*-3) [6]. Flaxseed oil is characterized by the highest content of ALA from plant sources and is known as a “superfood” for human health.

Numerous studies and reviews reported that flaxseed oil has played a significant role in preventing and alleviating cardiovascular diseases, hyperlipidemia, diabetes mellitus, hypertension, and cognitive dysfunction [5,6]. However, the health effects of flaxseed oil and whether they are determinable remain scientifically unproven. The uncertain physiological functions of flaxseed oil on human health can be attributed to the following: (1) some conclusions are summarized according to animal experiments or in vitro experiments; (2) limited numbers of human trials are not enough to prove that the functions of flaxseed oil are effective for most people; (3) unclearly distinguishing from various flaxseed products with different biological active ingredients; (4) some health benefits are directly contributed to *n*-3 polyunsaturated fatty acid EPA and DHA, which are two important metabolites with low biotransformation of ALA [7]; (5) confusion surrounding the function of flaxseed oil with that of linolenic acid from various foods. Therefore, it is necessary to provide a scientific and objective evaluation method to confirm most people’s health benefits of flaxseed oil. The GRADE (Grading of Recommendations Assessment, Development, and Evaluation) framework, widely adopted in evidence-based medicine, is a standardized methodology for evaluating evidence quality in systematic reviews, health technology assessments, and clinical guidelines, while also establishing the strength of healthcare recommendations [8,9]. Based on the GRADE approach, the Chinese Nutrition Society (CNS) established an evidence evaluation method for food and health. This evaluation framework comprises two key components: (1) evidence grading to categorize research quality, and (2) a systematic review of the evidence body. Through rigorous analysis of classified data, it determines whether a food significantly impacts specific human diseases or physiological functions. If the evaluation result of the evidence body is A or B, it means that the guiding practice drawn from this evidence body is credible or credible in most cases [10]. This evaluation approach has been applied to formulate and revise the Chinese Dietary Guidelines [11] led by the National Health Commission of the People’s Republic of China to clarify the relationship between food (such as whole grain, nuts, oils, fruits and vegetables, meat) and risk of diseases for further making scientific dietary recommendations for the public.

Basch et al. [12] assessed the scientific evidence regarding the health benefits of flaxseed and flaxseed oil using an evidence-grading approach comparable to the one mentioned above, yet less systematic and comprehensive. This result may have limited relevance today, mainly because early studies on the health effects of flaxseed oil in humans were insufficient to assess its efficacy. Moreover, the evidence evaluation method used has limitations. The objective of this study is to comprehensively search for research on the health effects of flaxseed oil on human health from 2001 to 2024 and to apply the food and health evidence evaluation method established by the CNS to accurately determine the health benefits of flaxseed oil for the human body.

## 2. Methods

### 2.1. Literature Search

The health benefits of flaxseed oil need to be determined by searching CNKI and Google Scholar databases, using “flaxseed oil OR flaxseed OR flaxseed product” and “bioactivity OR function”; reviews on flaxseed oil OR flaxseed OR flaxseed product are preferred. A literature search for each health benefit collected above was achieved in the medical databases of PubMed, Cochrane Library, Embase, Scopus, and CNKI from January 2001 to June 2024. The keywords “flaxseed oil OR α-linolenic OR flaxseed lignans” and “anti-inflammation OR inflammation”, “Type 2 diabetes OR T2DM OR non-insulin-dependent diabetes mellitus OR blood glucose OR glycosylated hemoglobin”, “cardiovascular diseases OR coronary disease OR heart disease”, “dementia OR Alzheimer OR cognition disorders OR cognition decline OR cognitive deficits OR cognitive impairment”, “hyperlipidemia OR hypercholesterolemia OR cholesterol OR atherosclerosis”, “blood pressure OR hypertension” were used.

### 2.2. Inclusion Criteria and Exclusion

Evidence was collected by the following steps: (1) animal studies, in vitro experiments, and investigations on molecular biology were excluded [10]; (2) systematic reviews with meta-analysis of randomized clinical trials were primarily chosen, and randomized clinical trial with a parallel was regarded as the second priority; (3) randomized controlled trials included in the systematic review evaluated were excluded; (4) randomized controlled trials with flaxseed oil intervention for at least two weeks and its dosage for the participants should be reported; (5) direct impact of flaxseed oil interventions on health outcomes; (6) the physiological indicators examined in the selected randomized controlled trials should directly affect health outcomes.

### 2.3. Evidence Quality Evaluation

**Figure 1:**
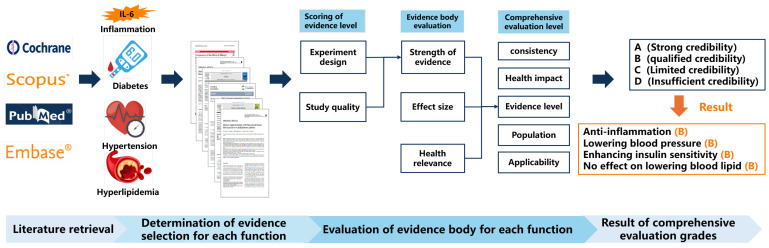
The flowchart of evidence evaluation.

The method of assessing the quality of evidence was carried out according to the “Food and Health Evidence-Based Review” [10] modified from the World Health Organization guidelines [8]. The specific evaluation process is shown in Figure 1. To begin with, each trial should be evaluated by the strength of evidence, effect size, and health relevance. Then, scoring the evidence refers to calculating the average for each trial selected, and marked as excellent (13–16), good (9–12), medium (8), and poor (1–4). Then, the evidence body containing the whole trials is comprehensively evaluated based on consistency, health impact, grade, population, and applicability, and ultimately graded as A, B, C, and D. If the body of evidence for one health benefit is rated as A or B, it indicates that flaxseed oil has these health effects on the human body. The process of evidence evaluation and detailed evaluation criteria are shown in Figure 1 and Supplementary Table S1(a–j), respectively.

## 3. Results

### 3.1. Literature Search and Screening

The potential health benefits of flaxseed oil on humans have been preliminary obtained by searching flaxseed oil-related reviews from CNKI and Google Scholar. A total of 2148 papers (Supplementary Table S2) were achieved from the search for each health benefit initially identified. Following the inclusion and exclusion criteria, thirteen papers were finally selected as evidence to evaluate.

### 3.2. The Health Benefits of Flaxseed Oil Grading by Evidence Evaluation

#### 3.2.1. Anti-Inflammation

The comprehensive evidence grade for flaxseed oil supplementation in reducing inflammatory cytokines is B (Table 1 and Table 2). Three papers (two systematic reviews and one randomized controlled trial) on the anti-inflammation of flaxseed oil were included based on the criteria of inclusion and exclusion. The results of two systematic reviews and one randomized controlled trial showed that a reduction in serum inflammatory cytokines and hypersensitive C-reactive protein (hs-CRP) was observed in participants intervened with flaxseed oil. A systematic review with meta-analysis showed that consuming flaxseed products significantly reduced inflammatory cytokine IL-6 (rather than TNF-α) in dyslipidemia patients [13]. The result of the flaxseed oil subgroup analysis further revealed that flaxseed oil (rather than whole flaxseed) played an important role in anti-inflammation, with reductions in IL-6 (−0.35 pg/mL, *p* = 0.033) and hs-CRP (−1.54 pg/mL, *p* = 0.004). Another systematic review found that flaxseed supplementation was associated with reduced C-reactive protein (CRP) and IL-6 levels, but not TNF-α. The subgroup analysis exhibited a significant effect on lowering the concentration of CRP in unhealthy or overweight participants administered lignan or whole flaxseed for over twelve weeks [14]. A retrospective study involving one hundred and twenty patients diagnosed with both type 2 diabetes mellitus and coronary heart disease from Hubei Province in China showed that hs-CRP in the flaxseed oil group supplemented with a dosage of 1000 mg/d was significantly lower than that in the control group after every three weeks during a fifteen-week intervention period (*p* < 0.05) [15].

#### 3.2.2. Lowering Blood Pressure

The comprehensive evidence grade for flaxseed oil supplementation in reducing blood pressure is B (Table 1 and Table 3). Three studies (two systematic reviews and one RCT) on the blood pressure-lowering effects of flaxseed oil were included as evidence based on the inclusion and exclusion criteria. The result of a systematic review with thirty-three randomized controlled trials and 2427 participants indicated that flaxseed supplementation had significant influence on reducing both systolic blood pressure (WMD: −3.19 mmHg; 95% CI: −4.15, −2.24, *p* < 0.001; I2 = 92.5%, *p* < 0.001) and diastolic blood pressure (WMD: −2.61 mmHg; 95% CI: −3.27, −1.94, *p* < 0.001; I2 = 94.1%, *p* < 0.001) [16]. Because of the high heterogeneity between studies, subgroup analysis was further conducted. Subgroup analysis revealed that flaxseed oil reduced systolic (WMD: −1.04 mmHg; 95% CI: −1.37, −0.72; *p* < 0.001) and diastolic blood pressure (WMD: −0.54 mmHg; 95% CI: −0.71, −0.38; *p* < 0.001), though its effect was weaker than whole flaxseed for diastolic pressure (WMD: −0.97 mmHg; 95% CI: −1.13, −0.82; *p* < 0.001) and lignans for systolic pressure (WMD: −2.12 mmHg; 95% CI: −3.86, −0.37; *p* = 0.017). Mahmudiono et al. [17] conducted a meta-analysis of RCTs to assess flaxseed oil’s impact on blood pressure in individuals with metabolic syndrome. This systematic review included five studies and demonstrated significant reduction in systolic blood pressure after flaxseed oil intake (WMD: −3.86 mmHg; 95% CI: −7.59, −0.13, *p* = 0.04). Nevertheless, there was no significant difference in diastolic blood pressure between the intervention group and the control group (WMD: −1.71 mmHg; 95% CI: −3.67, 0.26, *p* = 0.09). An RCT involving 87 middle-aged Greek men with dyslipidemia found that 12-week supplementation with flaxseed oil (8 g/d ALA) significantly reduced systolic (*p* = 0.016) and diastolic blood pressure (*p* = 0.011) compared to the control group (safflower oil, 11 g/d LA) [18]. Based on the results of the evidence stated above, dietary supplementation of flaxseed oil shows beneficial impacts on blood pressure for populations with dyslipidemia, hypertension, or other metabolic syndrome.

#### 3.2.3. Enhancing Insulin Sensitivity

The comprehensive evaluation grade of the evidence on improving insulin sensitivity with flaxseed oil supplementation is B (Table 1 and Table 4). Four studies (one systematic review and three RCTs) on the insulin sensitivity-enhancing effects of flaxseed oil were included as evidence based on the inclusion and exclusion criteria. The subgroup analysis in a systematic review [19] exhibited that in RCTs with ≥12-week interventions, sample sizes ≤ 50, and mean participant age ≤ 50 years, flaxseed oil significantly increased quantitative insulin sensitivity index (QUICKI) compared to whole flaxseed (WMD: 1.76; 95% CI: 0.82, 2.71; *p* < 0.001). A randomized controlled trial involving sixty patients with diabetic foot ulcers conducted by Soleimani et al. [20] showed that flaxseed oil supplementation (2000 mg/d) significantly improved QUICKI compared with the control group (*p* < 0.02). This finding aligns with an RCT administering flaxseed oil for 14 weeks to individuals with hyperglycemia and overweight [21]. Nevertheless, flaxseed oil supplementation with the same dosage as the abovementioned studies makes no significant effect on QUICKI for populations with normal blood glucose levels (*p* = 0.19) [22]. Based on the results of the evidence stated above, flaxseed oil supplementation may improve insulin sensitivity in for populations with dysglycemia.

#### 3.2.4. No Effect on Lowering Blood Lipid

The comprehensive evidence grade for flaxseed oil supplementation in blood lipid reduction is B (Table 1 and Table 5). Three papers (two systematic reviews and one randomized controlled trial) on the blood lipid reduction in flaxseed oil were included as evidence based on the inclusion and exclusion criteria. A systematic review by Yang et al. [13] analyzed nine RCTs (>500 participants with metabolic disorders, NAFLD, or hypertriglyceridemia) and found no significant effects of flaxseed oil on total cholesterol, triglycerides, low-density lipoprotein cholesterol (LDL-C), or high-density lipoprotein cholesterol (HDL-C). Hadi et al.’s review [23] of 32 RCTs (950 participants) reported no lipid improvements across populations with T2DM, CVD, hypercholesterolemia, or healthy status after 3–27 weeks of intervention. Rezaei et al.’s RCT [24] involving 68 obese NAFLD patients receiving 20 mL/d flaxseed oil for 12 weeks also showed no significant lipid changes versus the controls (sunflower oil). Compared with the control group (sunflower oil), flaxseed oil exerted no significant effect on total cholesterol, HDL-C, or LDL-C at the end of the intervention (*p* > 0.01). According to the evidence stated above, flaxseed oil intervention does not effectively reduce blood lipids in individuals with hyperlipidemia.

### 3.3. Uncertain Health Benefits of Flaxseed Oil

#### 3.3.1. Reducing Waist Circumference

A subgroup analysis in a systematic review was conducted using four randomized controlled trials involving 251 participants with overweight, metabolic syndrome, or dyslipidemia from Brazil, China, and Iran [13]. This subgroup analysis demonstrated that flaxseed oil consumption significantly reduced waist circumference compared with the control groups (WMD: −1.61 cm; 95% CI: −2.69 to −0.53). Another result from a subgroup analysis of a systematic review about flaxseed supplementation on anthropometric indices demonstrated that whole flaxseed supplementation—as opposed to flaxseed oil consumption—was associated with reduced waist circumference, particularly in patients with diabetes and polycystic ovarian syndrome who had a BMI > 30 kg/m^2^ and received interventions lasting over 20 weeks [25].

#### 3.3.2. Improvement of Mood and Cognition

A randomized controlled trial involving 51 American children and adolescents (aged 6–17 years) with symptomatic bipolar I or II disorder compared flaxseed oil (containing 6600 mg α-linolenic acid daily) to olive oil. Although no significant differences in mood symptom indicators were observed between groups after 16 weeks (*p* > 0.05), negative correlations were found between clinician-rated global symptom severity and final serum levels of α-linolenic acid (r = −0.45, *p* = 0.007) and eicosatetraenoic acid (r = −0.47, *p* = 0.005) [26]. Sixty Iranian women with depression (aged 18–45) participated in a randomized controlled trial conducted by Poorbaferani et al. [27], supplemented with flaxseed oil (2000 mg/d) for ten weeks. Compared to the control group (paraffin oil), the flaxseed oil group showed a significant increase in brain-derived neurotrophic factor concentration (1.12 ± 0.6 pg/mL vs. 0.2 ± 0.56 pg/mL; *p* < 0.0001) and a notable reduction in Beck Depression Inventory-II scores (−16.62 ± 7.03 vs. −8.45 ± 7.8; *p* < 0.0001) post-intervention. A randomized controlled trial by Ogawa et al. [28] involving 60 healthy Japanese older adults (aged 65–80 years) found that 12-week supplementation with flaxseed oil (2200 mg α-linolenic acid/day) significantly improved verbal fluency scores compared to the corn oil group (0.30 ± 0.53 vs. 0.03 ± 0.49; *p* < 0.05).

## 4. Discussion

We assessed the potential functions of flaxseed oil using the evidence-grading evaluation method, which demonstrated that flaxseed oil exhibits significant effects in anti-inflammatory responses, improving insulin sensitivity, and lowering blood pressure, but shows no significant effect on lowering blood lipids. In 2007, researchers applied the Jadad method (like the methodology used in this study) to evaluate evidence for 13 potential functions of flaxseed oil. The results showed that the evidence quality rating for each function was uniformly low, leading to the conclusion that existing research cannot confirm specific health benefits of flaxseed oil in humans [12]. This is primarily attributed to the limited number of human trials on flaxseed oil at the time, coupled with inadequate research quality. The poor study quality was mainly reflected in small sample sizes and unrepresentative study populations, such as trials exclusively involving postmenopausal women or male participants.

The meta-analyses included in the evidence conducted subgroup analyses on different flaxseed products (whole flaxseed, lignans, and flaxseed oil), with variations in their effects attributed to distinct bioactive compositions among these interventions. Flaxseed oil demonstrated significantly greater efficacy in reducing the inflammatory cytokine IL-6 and improving the QUICKI compared to whole flaxseed powder. However, it was less effective than both lignans and whole flaxseed powder in lowering CRP levels and showed inferior outcomes in blood pressure improvement relative to whole flaxseed powder. It can be inferred that ALA, the main component of flaxseed oil, has advantages in reducing inflammatory factors and improving insulin sensitivity.

In the RCTs included in the evidence, all participants underwent rigorous screening. Individuals with severe comorbidities, recent use of anti-inflammatory medications, regular long-term intake of nutritional supplements or flaxseed-related products, or consumption of fish exceeding 340 g per week were excluded. The body of evidence for each rated health outcome involved populations with broad diversity, spanning multiple countries across Europe, the Americas, Asia, and other regions, and included both sexes as well as young and elderly populations. These criteria aimed to minimize confounding factors and establish a clear causal relationship between flaxseed oil intake and health outcomes [18,20,21,22]. Regarding the method of administration, quantitative flaxseed oil supplementation was implemented under the premise of maintaining a regular diet. Daily dietary intake was designed to be balanced and was aligned with the respective national average levels.

Inflammatory cytokines are low-molecular-weight soluble proteins responsible for the initiation and mediation of inflammatory responses and are regarded as biomarkers in many clinical disorders such as non-communicable diseases, cancer, type 2 diabetes, and cardiovascular diseases [29,30,31]. Based on a comprehensive evaluation of evidence gradation demonstrating the anti-inflammatory efficacy of flaxseed oil, it can be inferred that flaxseed oil intervention can alleviate the progression of non-communicable chronic diseases or inhibit their development by reducing inflammatory cytokines [32]. IL-6, IL-8, IL-12, and TNF-a are principal pro-inflammatory cytokines that amplify the progression of inflammation and are abundantly distributed in patients with severe disease conditions [31,33,34]. Yang et al. [13] speculated that α-linolenic acid as the main component of flaxseed oil inhibited the synthesis of arachidonic acid and then decreased its level in tissues by competing with linoleic acid in the same metabolic pathway to produce long-chain fatty acids. The absence of arachidonic acid could reduce pro-inflammatory cytokines and suppress the inflammatory progression. Researchers have found that serum *n*-3 polyunsaturated fatty acids were inversely associated with hs-CRP levels [35]. It may be concluded that flaxseed oil supplementation is beneficial for preventing the progression of inflammation and the onset of diseases.

High blood pressure is highly related to cardiovascular diseases and all-cause mortality [36]. The evidence stated above showed that flaxseed oil consumption regulates blood pressure in populations with metabolic syndrome, hyperlipidemia, or hypertension from different countries. Li et al. [37] demonstrated that α-linolenic acid (ALA) exerts antihypertensive effects in spontaneously hypertensive rats. a-linolenic acid may improve endothelial function and alleviate hypertension by counteracting the decline of SIRT3 function, to restore autophagy and mitochondrial redox balance in endothelial cells. Ogawa et al. [38] demonstrated that dietary α-linolenic acid inhibits the activity of angiotensin-converting enzymes and significantly reduces the mRNA expression levels of related inflammatory markers. Another study demonstrated that oral supplementation with α-linolenic acid reduces blood pressure through bradykinin-stimulated increases in prostaglandin I_2_ and nitric oxide levels, which enhance blood flow and promote vascular smooth muscle relaxation, ultimately lowering blood pressure [39]. Some researchers also suggested that flaxseed oil supplementation alleviates hypertension through anti-inflammation, which is highly related to cardiovascular diseases and improves the elasticity of arterials [40,41,42].

Type 2 diabetes mellitus (T2DM) is a chronic disease with high blood glucose levels accompanied by disordered insulin secretion, low insulin sensitivity, and high insulin resistance [43,44]. The quantitative insulin sensitivity check index (QUICKI) is a clinical indicator for estimating insulin sensitivity and assessing insulin resistance in T2DM patients or those at risk, calculated using fasting blood glucose and insulin levels [45]. Preserved endothelial insulin signaling and balanced insulin–aldosterone levels enhance nitric oxide availability, reducing vascular stiffness and cardiovascular disease risk [46,47]. Yu et al. [48] demonstrated that α-linolenic acid ameliorates hepatic insulin resistance by restoring mitochondrial quality control networks. This effect is attributed to its specific localization in the mitochondrial membranes of hepatocytes from obese mice fed a lard-based high-fat diet, concomitant with upregulated expression of SIRT1, PPARγ coactivator-1α (PGC-1α), and extracellular signal-regulated kinase (ERK). Researchers also assume that the mechanism of enhancing insulin sensitivity is related to diminishing circulating lipids, scavenging free radicals, and reducing inflammatory cytokines directly influenced by α-linolenic acid [49,50,51].

Current evidence suggests that flaxseed oil has no significant impact on total cholesterol, triglycerides, HDL-C, or LDL-C in hyperlipidemic patients. However, the results of subgroup analysis from two systematic reviews [13,23] indicated that whole flaxseed supplementation exhibited a significant lowering effect on total cholesterol, total triglyceride, and LDL-C, and flaxseed lignan intake decreased total cholesterol in hypercholesterol and hyperlipidemia individuals. Flaxseed can be regarded as a great source of dietary fiber including soluble fiber and insoluble fiber, accounting for 35–45% of the whole seed. The soluble fiber can increase intraluminal viscosity by interfering with bile acid metabolism, reduce lipid absorption through inhibiting micelle formation, or impede cholesterol synthesis in the liver to delay the reabsorption of bile acid [52,53]. Previous studies have found that secoisolariciresinol diglycoside, the dominant lignan in flaxseed, prevents cholesterol synthesis and secretion by inhibiting the gene expression of acetyl-CoA acetyltransferase 2 in the liver and by modulating the endoplasmic reticulum stress/Ca^2+^/ER-mitochondrial axis [54,55]. Moreover, the insignificant effect of flaxseed oil on reducing blood lipid levels may be due to the fact that the control group received interventions containing monounsaturated fatty acids and omega-6 polyunsaturated fatty acids, which themselves also have lipid-lowering properties [56]. Researchers have also inferred that the lowering of blood lipids contributed to the synergistic effect of α-linolenic acid, dietary fiber, lignan, and probably other components (such as bioactive peptides) in flaxseed [13,23].

The health benefits of flaxseed oil discussed in the “Uncertain Health Benefits of Flaxseed Oil” section of this paper are inconclusive due to insufficient evidence, according to the Food and Health Evidence Evaluation Methods [10]. Yang et al. [13] reported that flaxseed oil consumption showed a significant reduction in waist circumstance, which is used to clinically assess the amount of visceral fat correlated with CVD risk [57]. Also, the subgroup analysis indicated that whole flaxseed intervention significantly reduced the waist-to-height ratio, which is an indicator to measure the degree of obesity [58]. Similarly, Musazadeh et al. [25] analyzed that flaxseed oil showed no effect on waist circumstances for people with obesity and diabetes after twelve-week intervention, but whole flaxseed did. Another systematic review also showed that whole flaxseed is an effective strategy for managing weight, especially for obese individuals looking to reduce their weight [59]. The main component of dietary fiber from whole flaxseed (rather than flaxseed oil) accelerates the basic metabolism, inhibits fat accumulation, and significantly increases the abundance of g_Akkermansia and g_Bifidobacterium, which are gut microbiota negatively related to obesity [60]. Dietary fiber intake promotes the secretion of short-chain fatty acids, which activates free fatty acid receptors 3 and 4, thereby facilitating the release of the anorectic gut hormone peptide YY and ultimately inducing appetite suppression in humans [61]. ALA is one of the energy-providing nutrients for humans, and its effect on waist circumference or fat accumulation remains to be investigated according to sufficient evidence. Moreover, as a multifactorial chronic disease, obesity requires multimodal therapeutic strategies beyond dietary modification. A comprehensive systematic review and meta-analysis of 149 RCTs demonstrated that exercise training induces clinically significant weight reduction, visceral fat loss in obese populations [62]. Though the effect of flaxseed oil consumption on waist circumstances is still unclear, compared with saturated fatty acid or *n*-6 polyunsaturated fatty acids enriched oil, it is clearly a good choice for people with obesity [63].

The physiological functions of DHA in the brain are highly associated with its unique molecular structure to facilitate the quantum transfer and communication of π-electrons, clarifying the greater ability to manage and coordinate highly intelligent neural signals possessed by DHA rather than EPA [64,65]. It is reported that alpha-linolenic acid can be biologically converted to long-chain *n*-3 polyunsaturated fatty acids EPA and DHA [6]. However, the ratio of ALA converted into DHA is so limited that it cannot adequately satisfy the physiological needs of human adults [7,66]. Importantly, the brain maintains DHA levels primarily through absorption from exogenous lipid sources rather than endogenous biosynthesis, a process shown to be less efficient in the brain than in other body tissues [67,68,69]. These metabolic limitations suggest that the direct dietary intake of DHA-rich foods provides more efficient support for cognitive enhancement than indirect strategies relying on endogenous ALA conversion.

## 5. Strengths and Limitations

The strength of this study is that it first makes reliable health benefits of flaxseed oil supplementation according to the food and health evidence evaluation method based on human studies from 2001 to 2024. Systematic review refers to a scientifically rigorous literature review method. It aims to systematically collect, assess, and synthesize all relevant studies on a health-related issue through standardized evaluation methods to make clinical decisions. For assessing the health outcomes of flaxseed oil, systematic reviews and RCTs form the evidence hierarchy used to grade outcomes and establish a robust body of evidence, ensuring reliable evaluation. Furthermore, while current evidence for certain effects remains inconclusive, these findings may be definitively confirmed as future human studies on flaxseed oil’s health impacts emerge.

## 6. Conclusions

The health impacts of flaxseed oil consumption are identified according to Food and Health Evidence Evaluation Methods. The results of the evidence body evaluation show that flaxseed oil intervention has significant effects on anti-inflammation, lowering blood pressure, and enhancing insulin sensitivity while exerting no obvious influence on blood lipid improvement. In addition, waist circumference reduction and brain neural regulation are uncertain because there is still not enough human trial evidence to evaluate.

## Figures and Tables

**Table 1:** Comprehensive grade of evidence body for diseases.

Disease	Comprehensive Grade		Level of Evidence	Consistency	Health Impact	Study Population	Applicability
Inflammation	B	**Grade**	Good	Good	Good	Good	Good
		**Remarks**	Two systematic reviews and one retrospective study (average score 12.8)	More than 70% of the studies are consistent	Flaxseed oil has a significant effect on improving inflammation.	Including Europe, America, Asia, and other countries	Suitable for most people
Hypertension	B	**Grade**	Good	Good	Excellent	Good	Good
		**Remarks**	Two systematic reviews and one RCT (average score 12.6)	More than 70% of the studies are consistent	All study results show that intake of flaxseed oil can reduce blood pressure.	Including Europe, America, Asia, and other countries	Suitable for most people
Type 2 diabetes mellitus	B	**Grade**	Good	Good	Good	Good	Good
		**Remarks**	One systematic review and three RCTs (average score 12.5)	More than 70% of the studies are consistent	More than 70% of the study results show that the intake of flaxseed oil increases insulin sensitivity.	Including Europe, America, Asia countries	Suitable for most people
Hyperlipidemia	B	**Grade**	Good	Good	Poor	Good	Good
		**Remarks**	2 systematic reviews and 1 RCT (average score 9.3)	More than 70% of the studies are consistent	All results showed that flaxseed oil has **no significant effect** on reducing blood lipid.	Including Europe, America, Asia countries	Suitable for Chinese, but with some exceptions

**Table 2:** Evidence body on flaxseed oil and inflammatory cytokines.

	Yang et al. [13]	Askarpour et al. [14]	Jiang et al. [15]
Study Type	In a systematic review, nine related RCTs are included in the subgroup.	In a systematic review, twenty-one related RCTs are included in the subgroup.	A retrospective study.
Investigation Method	4–13 week intervention experiments.	3–18 week intervention experiments.	A 15-week intervention experiment.
Number of Cases	630	560	120
Participants’ Characteristics	Dyslipidemia populations from China, Germany, Canada, Iran, Greece, and Brazil, male and female, 45–66 years old.	Populations from Australia, Iran, Brazil, America, Germany, Canada, Greece, and Finland, male and female, 24–68 years old.	Populations diagnosed with both T2DM and CHD from Hubei, China, male and female, 40–100 years old.
Intake Amount/Frequency	Intake of flaxseed oil at a dosage of 0.4 g–24 g/day, with the control group consuming soybean oil, corn oil, and sunflower oil for 4 to 13 weeks.	Intake of flaxseed oil containing 1.0–13.7 g/d of ALA, with the control group consuming sunflower oil, soybean oil, medium-chain fatty acids, olive oil, etc., over 3–18 weeks.	Intake of flaxseed oil at a dosage of 1.0 g/day, with the control group taking 1.0 g/day of paraffin oil, for 15 weeks.
Results	Reduction in IL-6 (−0.35 pg/mL, *p* = 0.033) and hs-CRP (−1.54 mg/L, *p* = 0.004).	For unhealthy or overweight populations, the intake of flaxseed oil for less than 12 weeks can reduce IL-6 by −0.268 (−0.393, −0.143), but does not affect reducing CRP and TNF-α.	There was a significant difference in hs-CRP (*p* = 0.02) in the flaxseed oil group, compared with the control group.
Impact on Risk	Protective	Protective	Protective

**Table 3:** Evidence body on flaxseed oil and blood pressure.

	Li et al. [16]	Mahmudiono et al. [17]	Paschos et al. [18]
Study Type	In a systematic review, eleven related RCTs are included in the subgroup.	In a systematic review, five related RCTs are included in the subgroup	RCT
Investigation Method	4–24 week intervention experiments.	6–12 week intervention experiments	A 12-week intervention experiment
Number of Cases	734	117	87
Participants’ Characteristics	Both healthy and unhealthy people from Finland, Canada, Australia, America, and Iran, male and female, 32–63 years old.	People with metabolic syndrome, hypercholesterolemia, and hypertension from Iran, the United States, Australia, China, and Greece, male and female, 49–56 years old	Dyslipidemia populations from Greece, male, 52–55 years old
Intake Amount/Frequency	Intake of flaxseed oil at a dosage of 1–30 g/day, with the control group consuming soybean oil, corn oil, sunflower oil, and hempseed oil for a period of 4 to 24 weeks.	Flaxseed oil was consumed at a dosage of 2.2–23 g/day, with the control group taking soybean oil, corn oil, sunflower oil, and safflower oil for a duration of 6 to 12 weeks	Consumption of 15 mL of flaxseed oil containing 8 g/day of ALA, with the control group consuming 15 mL of safflower oil containing 11 g/day of LA, over 12 weeks
Results	Subgroup analysis showed that flaxseed oil could reduce systolic blood pressure by a WMD of −1.04 (95% CI: −1.73, −0.72) and diastolic blood pressure by a WMD of −0.54 (95% CI: −0.71, −0.38).	Flaxseed oil could significantly reduce systolic blood pressure by −3.86 mmHg (−7.59, −0.13), but had no significant effect on the reduction in diastolic blood pressure compared to the control group	Flaxseed oil significantly reduced systolic blood pressure (*p* = 0.016) and diastolic blood pressure (*p* = 0.011)
Impact on Risk	Protective	Protective	Protective

**Table 4:** Evidence body on flaxseed oil and insulin sensitivity.

	Kavyani et al. [19]	Soleimani et al. [20]	Hajiahmadi et al. [21]	Jamilian et al. [22]
Study Type	In a systematic review, seven related RCTs are included in the subgroup.	RCT	RCT	RCT
Investigation Method	6–12 week intervention experiments.	12-week intervention experiment	14-week intervention experiment	12-week intervention experiment
Number of Cases	417	60	36	40
Participants’ Characteristics	People with T2 DM, metabolic disorders, and non-alcoholic fatty liver from the USA, Canada, and Iran, including both men and women, 30–65 years old.	An Iranian population with diabetic foot ulcers, including both men and women, is around 40–85 years old	An Iranian population with overweight and prediabetes, including both men and women, around 40 years old	An Iranian population with endometrial hyperplasia but with normal blood sugar levels, women 44–47 years old
Intake Amount/Frequency	Intake of flaxseed oil at a dosage of 2.0–30.0 g/day, with the control group consuming paraffin oil and sunflower oil for 6 to 12 weeks.	Intake of 2.0 g/day flaxseed oil, with the control group consuming paraffin oil over 12 weeks	Intake of 2.0 g/day flaxseed oil, with the control group consuming paraffin oil over 14 weeks	Intake of 2.0 g/day flaxseed oil, with the control group consuming paraffin oil over 12 weeks
Results	Compared with the control group, QUICKI was significantly increased by WMD 1.76 (95% CI: 0.82, −2.71), (*p* < 0.001).	Compared with the control group, QUICKI was significantly increased (*p* < 0.02)	Compared with the control group, QUICKI was significantly increased (*p* < 0.001)	Compared with the control group, no significant differences in QUICKI between groups. However, fasting blood glucose was significantly decreased (*p* = 0.003)
Impact on Risk	Protective	Protective	Protective	Unprotective

**Table 5:** Evidence body on flaxseed oil and blood lipid.

	Yang et al. [13]	Hadi et al. [23]	Rezaei et al. [24]
Study Type	A systematic review, nine related RCTs are included in the subgroup.	A systematic review, thirty-two related RCTs are included in the subgroup.	RCT
Investigation Method	4–13 week intervention experiments.	3–27 week intervention experiments.	12-week intervention experiment
Number of Cases	554	950	68
Participants’ Characteristics	Participants with T2DM, metabolic disorders, non-alcohol fatty liver, and hypertriglyceridemia from China, Canada, Germany, Greece, and Iran, including both men and women, 47–64 years old.	Participants with T2DM, CVD, hypercholesterolemia, and healthy people from Canada, Brazil, Germany, USA, Japan, Australia, Greece, Finland, the UK, and China, including men and women between 25 and 68 years old.	Iranian obesity patients with non-alcoholic fatty liver disease, including both men and women, 40–45 years old
Intake Amount/Frequency	Intake of flaxseed oil at a dosage of 2.0–25.0 g/day, with the control group consuming paraffin oil, sunflower oil, corn oil, safflower oil, and high-oleic canola oil for a period of 4 to 13 weeks.	Intake of flaxseed oil containing 1.0–21.0 g/day ALA, with the control group consuming paraffin oil, sunflower oil, corn oil, safflower oil, olive oil, and hempseed oil over a period of 3–27 weeks.	Intake of 20 mL/day of flaxseed oil, with the control group consuming sunflower oil over a period of 12 weeks
Results	Flaxseed oil intervention had no significant effect on total cholesterol, triglyceride, LDL-C, or HDL-C.	Flaxseed oil intervention had no significant effect on total cholesterol, triglyceride, LDL-C, or HDL-C.	There was no significant change in total cholesterol, HDL-C, or LDL-C compared with the control group (*p* > 0.01)
Impact on Risk	Unprotective	Unprotective	Unprotective

## Data Availability

The original contributions presented in this study are included in the article. Further inquiries can be directed to the corresponding authors.

## Supplementary Materials

The following supporting information can be downloaded at: https://www.mdpi.com/article/10.3390/nu17111791/s1, Table S1: (a) Grading and scoring of experimental design, (b) Quality evaluation of randomized clinical trial and cohort study, (c) Effect size grading criteria, (d) Health relevance of evidence, (e) Levels of evidence, (f) Levels of evidence consistency, (g) Classification of health impact levels, (h) The classification of the similarity grade between the study population and the target population, (i) Levels of applicability, (j) Comprehensive evaluation grades and criteria; Table S2: Comprehensive evaluation grades and criteria.
