# Supplementary material for: The Impact of Flaxseed (Linum usitatissimum L.) Oil Supplementation on Human Health: A Human-Centric Evidence-Graded Approach
Source: Nutrients. 2025 May 25;17(11):1791. doi: 10.3390/nu17111791 (PMC12157800; doi:10.3390/nu17111791)
Supplement: Supplementary file 1 [file nutrients-17-01791-s001.zip › nutrients-3619840-supplementary.pdf]

Table S1. (a) Grading and scoring of experimental design.

**Grade.**	Experiment design	Score
I	A systematic review of grade II trial (or Meta-analysis)	4
II	Randomized clinical trials	3
III-1	Quasi-randomized control trials	2
III-2	Non-randomized comparative studies and systematic reviews of these studies.<br>(Example: a cohort study, a case-control study)	2
III-3	Non-synchronized comparative studies without concurrent controls	2
IV	Case series with only treatment outcomes and case series with pre-and post-treatment comparisons<br>(Example: Cross-sectional study)	1

Table S1. (b) Quality evaluation of randomized clinical trial and cohort study.

**Experiment design.**	Category	Trial quality	Score
Randomized clinical trial	Sample size	Big (group>50 participants)	1
		Small (group<50 participants)	0
	Blind method	Double-blind (or triple-blind)	2
		Singe blind	1
		No blind	0
	Dropout rate	⩽20%	1
		> 20%	0
	Intervention time	⩾Two months	1
		< Two months	0
Cohort study	Incidence rate	Big (exposure group exceeded 100/person-year)	1
		Small (exposure group under 100/person-year)	0
	Blind method	Blind	1
		No-blind	0
	Dropout rate	⩽20%	1
		> 20%	0
	Confounding factors	Control	1
		No-control	0
	Follow-up time	⩾ 2 months	1
		< 2 months	0

Notes: Since the evidence included in this study mainly consists of randomized clinical trials and a retrospective study that belonged to a cohort study, the quality assessment methods for the case-control and cross-sectional studies were not described in the supplemental data.

Table S1. (c) Effect size grading criteria.

**Effect size.**	Score
The results are statistically significant, and the entire confidence interval has clinical significance.	4
The results are statistically significant, but the confidence interval includes values that are not clinically meaningful.	3
The results are statistically significant, but the entire confidence interval does not have clinical significance.	2
The results are not statistically significant, but the confidence interval includes clinically meaningful values.	1
The results are not statistically significant.	0

Table S1. (d) Health relevance of evidence.

Outcome type	Score
Patient-related outcomes.	3
Health outcomes	2
Surrogate outcomes	1

Notes: Patient-related outcomes refer to the comprehensive evaluation of a patient's subjective feelings, functional status, and quality of life. Health outcomes refer to the clinical events that have the most significant impact on patients and are most wanted to be avoided, commonly including death and sudden death, stroke, and acute myocardial infarction. Surrogate outcomes refer to physiological indicators that can fully reflect the direct impact of interventions on health outcomes, such as cholesterol, blood glucose, and blood pressure.

Table S1. (e) Levels of evidence.

Research evidence	Average score	Level
Evidence body (including all evidence)	13～16	Excellent
	9～12	Good
	5～8	Medium
	1～4	Poor

Table S1. (f) Levels of evidence consistency.

Level	Consistency
Excellent	All studies are consistent
Good	More than 70% of the studies are consistent
Medium	50%～70%of the studies are consistent
Poor	Less than 50% of the studies are consistent

Notes: Consistency refers to the similarity or uniformity of the essential characteristics or features of all studies included in the body of evidence.

Table S1. (g) Classification of health impact levels.

Level	Health impact
Excellent	All study results consistently show that a certain food impacts health.
Good	70% of the study results are consistent in showing that a certain food has an impact on health.
Medium	50%～70% of the study results are consistent in showing that a certain food has an impact on health.
Poor	Less than 50% of the study results are consistent in showing that a certain food has an impact on health.

Table S1. (h) The classification of the similarity grade between the study population and the target population.

Level	Population
Excellent	The population constituting the evidence body is consistent with the target population.
Good	The population constituting the evidence body is similar to the target population.
Medium	The population constituting the evidence body differs from the target population but with relatively small racial and age differences and can be reasonably applied.
Poor	The population constituting the evidence body is different from the target population, making it difficult to determine whether it is reasonably applicable to the target population.

Notes: The target population for the evidence evaluation method for food and health established by the Chinese Nutrition Society is the Chinese people (CNS, 2016), while the majority of the world's population is regarded as the target population in this study.

Table S1. (i) Levels of applicability.

Level	Applicability
Excellent	Directly applicable to the target population
Good	Applicable to the target population, but with individual precautions
Medium	Applicable to the target population, but there are many precautions
Poor	Not applicable to the target population

Notes: The target population for the evidence evaluation method for food and health established by the Chinese Nutrition Society is the Chinese people (CNS, 2016), while the majority of the world's population is regarded as the target population in this study.

Table S1. (j) Comprehensive evaluation grades and criteria.

Grade	Description	Evaluation criteria
A	The conclusion drawn from this evidence body is credible to guide practice.	5 excellent
B	In most cases, the conclusion drawn from this evidence body is credible to guide practice.	3～5 excellent or good
C	The conclusion drawn from this evidence body has a certain degree of credibility in guiding practice, but caution should be exercised when applying it.	1～2 excellent or good
D	The conclusion drawn from the evidence body is weak in guiding practice, so it must be used with extreme caution or not use it at all.	No one is excellent or good

Table S2. Comprehensive evaluation grades and criteria.

Diseases	Keywords	Number		Total
		Chinese	English	
Inflammation	Flaxseed oil，α-linolenic，flaxseed lignans，anti-inflammation，inflammation	0	373	373
Type II Diabetes Mellitus	*Type 2 diabetes, type 2 diabetes mellitus, T2D, T2DM, NIDDM, non-insulin-dependent diabetes mellitus, blood glucose, glycosylated hemoglobin	17	253	280
Cardiovascular disease	* cardiovascular diseases OR coronary disease OR heart disease	3	651	654
Dementia and Cognitive Impairment	*dementia, Alzheimer's, cognition disorders, cognition decline, cognitive deficits, cognitive impairment	0	198	198
Hyperlipidemia	*hyperlipidemia, hypercholesterolemia, cholesterol, atherosclerosis	2	315	317
Hypertension	*blood pressure, hypertension	2	334	336
Total		24	2124	2148

*represents Flaxseed oil, α-linolenic, flaxseed lignans.
